# Thymoma with systemic lupus erythematosus and immune-related anemia: A case of thymoma with SLE and IRA

**DOI:** 10.1097/MD.0000000000032077

**Published:** 2022-12-09

**Authors:** Huayang Zhang, Ting Chen, Xuesong Zhang, Peng Zhang, Yuan Chen

**Affiliations:** a Department of Cardiothoracic Surgery, Tianjin Medical University General Hospital Airport Hospital, Tianjin, China; b Department of Pharmacy, Tianjin First Center Hospital, Tianjin, China; c Department of Cardiothoracic Surgery, Tianjin Medical University, Tianjin, China; d Department of Cardiothoracic Surgery, Tianjin Medical University General Hospital, Tianjin, China; e Department of Cardiothoracic Surgery, Tianjin Medical University General Hospital, Tianjin, China.

**Keywords:** autoimmunity, SLE, thymectomy, thymomas, thymus

## Abstract

Thymic epithelial tumors (TETs), including thymomas and thymic carcinomas. Thymomas are rare tumors, but are one of the most common mediastinal neoplasms in adults. The pathology of thymoma with Systemic Lupus Erythematosus (SLE) and severe pancytopenia is rarely reported. Thus, the current study demonstrates a case of thymoma with SLE and immune-related anemia with unique clinical manifestation.

**Patient concerns:**

A 27-year-old woman suffered from abdominal pain, arthralgia, intermittent high fever for a long time.

**Diagnosis:**

Based on the clinical and histopathological manifestations, diagnosis of thymoma with SLE and immune-related anemia was established.

**Interventions:**

Patient was treated with methylprednisolone and a complete thymectomy and thymomectomy, the CAP regimen was given 4 times of adjuvant chemotherapy after the operation.

**Outcomes:**

After inter-disciplinary consultation as well as extensive discussion and steroid pulse therapy underwent surgery, the patient’s blood count and immune function gradually entry sent back to normal.

**Conclusion:**

we present the diagnosis and treatment of a case of thymoma with SLE and immune-related anemia, and provides references for the clinical diagnosis and treatment of thymoma combined with SLE, and attempts to explain that SLE patients with thymoma may contribute to the clinical remission of SLE after thymoma resection. It should arouse the attention of clinicians when diagnosing and treating related diseases.

## 1. Introduction

Thymic epithelial tumors (TETs), including thymomas and thymic carcinomas.^[1]^ Thymomas are rare tumors, only accounts for 0.2% to 1.5% of cancer, but are one of the most common mediastinal neoplasms in adults account for 25% of mediastinal tumors.^[2]^ Thymomas have been associated with paraneoplastic autoimmune syndromes more frequently than have thymic carcinomas, including myasthenia gravis, Systemic lupus erythematosus (SLE), pure red cell anemia, pemphigoid, polymyositis, etc.^[3,4]^ The degree of prognosis is related to the world health organization (WHO) classification and Masaoka staging of thymoma.^[1,3]^ SLE associated with thymoma accounts for 1.5% of all related autoimmune diseases.^[3]^

SLE is a chronic multi-organ debilitating autoimmune disease, which mainly afflicts women in the reproductive years. Anemia is a common clinical manifestation of SLE patients. Most anemias are chronic anemia,^[5]^ autoimmune hemolytic anemia, iron deficiency anemia. Anemic of chronic disease in SLE patients is usually caused by the insufficient production of erythropoietin (Epo) and RBC resistance to Epo; lupus nephritis can cause kidney disease in SLE patients Functional failure, which in turn leads to insufficient Epo and the presence of anti-Epo antibodies.^[6]^ aCL-IgG and/or aCL-IgM are frequently present in immune-related anemia patients, indicating that these antibodies may be in autoantibodies It plays a major role in the pathogenesis of inducing red blood cell destruction. In addition, Immune-Related Anemia is closely linked to low complement levels and the presence of anti-dsDNA antibodies.^[7]^

The pathology of thymoma with SLE and severe pancytopenia is rarely reported. This article reports a case of thymoma with SLE with pancytopenia as the highest manifestation, which was improved and discharged after treatment. This case had severe reduction of whole blood cells, complicated spleen, huge cystic and solid images of the anterior mediastinum, and was initially misdiagnosed as lymphoma.

## 2. Case report

**Figure 1.**
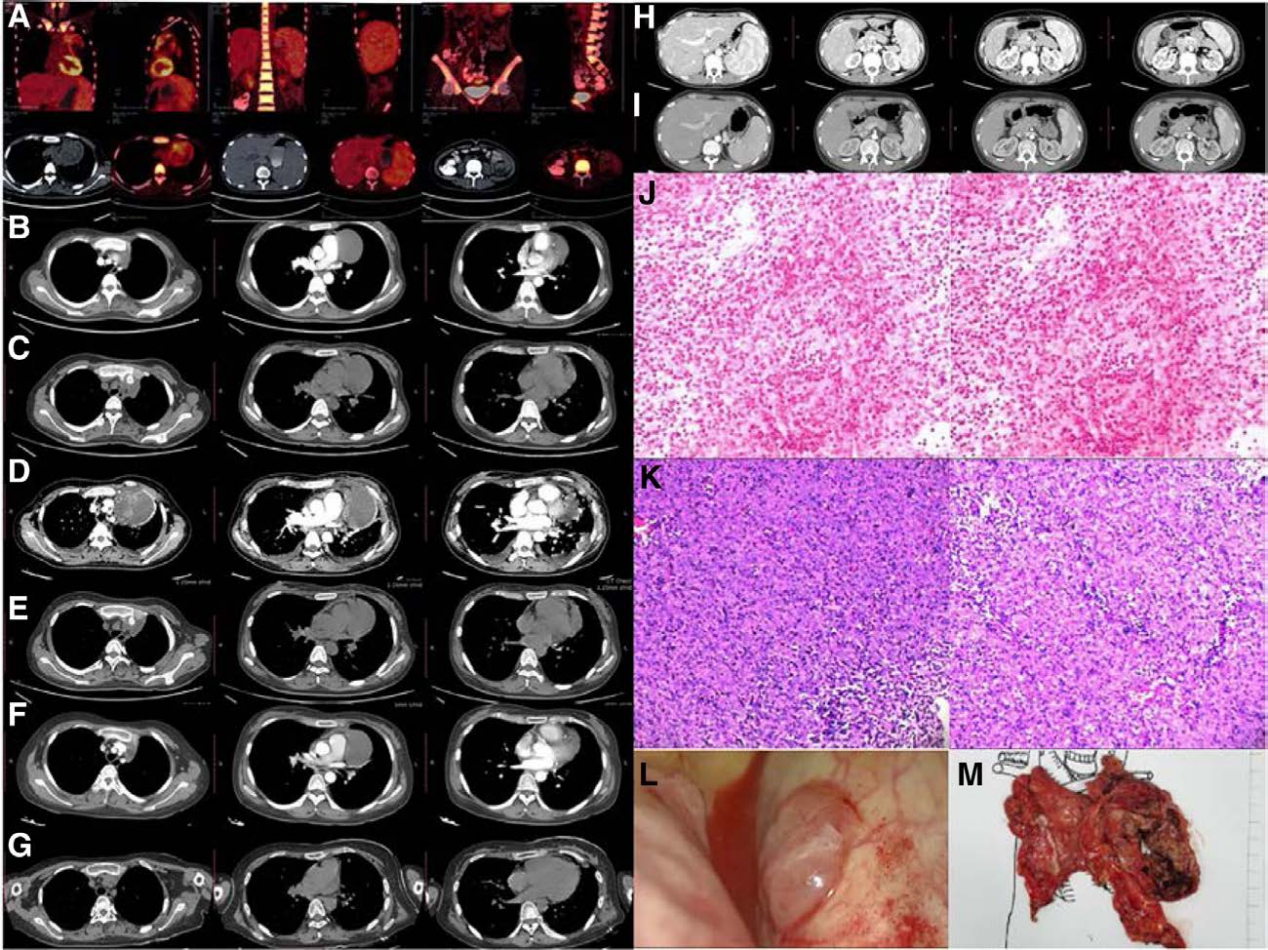
The PET/CT of the patient, huge anterior mediastinum mass, increased metabolism, secondary pericardial effusion and left pleural effusion, enlarged lymph nodes in the hepatic portal and retroperitoneum with increased metabolism, enlarged spleen with active metabolism (A). Evolution of the chest CT of the patient during the Tianjin Medical University General Hospital, September 29, 2020, the chest CT revealed that the anterior mediastinal mass was a multilocular cystic solid mass with a maximum cross-sectional area of about 6.5 × 7.0 cm (B). October 27, 2020, the chest CT revealed that the anterior mediastinal multilocular cystic solid mass was slightly larger than the previous one, and the largest cross-sectional area was about 6.6 × 7.2 cm (C). January 5, 2021, the chest CT revealed that the multilocular cystic solid mass was significantly larger than before, with a maximum cross-sectional area of about 13.1 × 8.1 cm, with clear boundaries. The mass surrounded the main pulmonary artery and locally protruded into the upper left lung leaf (D). January 22, 2021, the chest CT revealed that the multilocular cystic solid mass was smaller than before, and the size of the patient’s anterior mediastinum mass was about 8.1 × 6.0 cm (E). February 19, 2021, the chest CT revealed that the maximum cross-sectional area was about 8.1 × 6.0 cm (F). March 9, 2021, the chest CT revealed that the patient’s chest condition after surgery (G). Evolution of the CT of abdomina the patient during the Tianjin Medical University General Hospital on January 5, 2021 (H) and February 19, 2021 (I). Thymoma biopsy (J, K). During the operation, several nodules were found in the chest wall and diaphragm muscle (L) and resected tumor (M). CT = computed tomography, PET/CT = positron emission tomography/ computed tomography.

We describe a case of thymoma with SLE and severe pancytopenia in our center in June 2020. The patient suffered from pain—abdominal pain, arthralgia, intermittent high fever—for a long time until she came to our center. Clinical evaluation was made in A hospital in Beijing (Fig. 1A).

**Figure 2.**
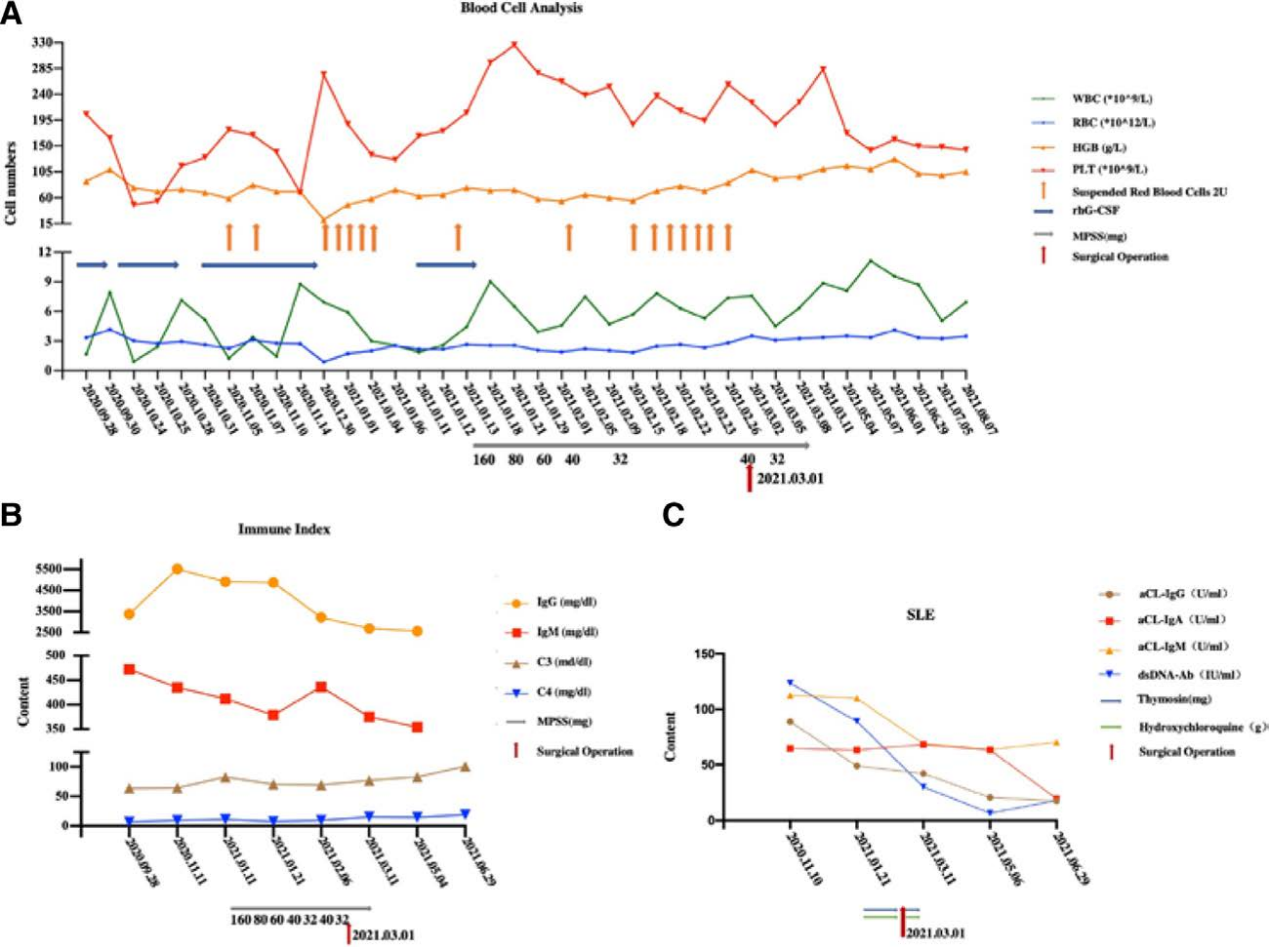
The blood cell analysis, immune index and SLE of the patient of Tianjin Medical University General Hospital. The patient’s WBC, RBC, HGB, PLT levels of the patient of Tianjin Medical University General Hospital (A). The patient’s IgM, IgG, C3, C4 levels of the patient of Tianjin Medical University General Hospital(B). The paitent’s aCL-IgG, aCL-IgA, aCL-IgM, dsDNA-Ab levels of the patient of Tianjin Medical University General Hospital (C). SLE = systemic lupus erythematosus.

The patient went to our center in September 2020, and the spleen was checked over the level of the umbilical cord. After the serology and bone marrow biopsy, the results showed that the granule system increased, the erythroid system was reduced, the megakaryocyte hyperplasia, low bone marrow hyperplasia. The patient was evaluated by multi-disciplinary treatment, it is considered that the patient with SLE combined with lymphoma is feasible for surgical treatment. But the patient’s general condition suggest that the patient’s immune disease is active, the operation risk—bleeding, embolism, and even death during the operation—is high. After 3 months, the patient had intermittent right upper abdominal pain. She went to the hospital again for laboratory-related examinations and flow cytometry (Table 1). The blood routine showed severe anemia and immune-related indicators are abnormal (Fig. 2). The medical image revealed splenomegaly (Fig. 1H) and the anterior mediastinum was significantly larger than the previous multilocular cystic solid mass (Fig. 1D). At this time, the patient’s anterior mediastinum tumor was significantly enlarged, and the solid component could be punctured. Pathology from a computed tomography (CT)-guided biopsy revealed tended to be type B2 thymoma (Fig. 1J). The patient was evaluated by multi-disciplinary treatment, it is recommended that SLE be controlled first, and then surgical treatment. After the treatment of methylprednisolone, the immune index approaches normal, and the hemoglobin is close to normal. Most importantly, the anterior mediastinum mass is significantly reduced (Fig. 1E–G) compared to the anterior mediastinum before treatment (Fig. 1B–D), the spleen is smaller (Fig. 1I) than before treatment (Fig. 1H). In March 2021, our center reevaluated the patient and subsequently performed a complete thymectomy and thymomectomy, intraoperative exploration also revealed 3 metastatic nodules on the left chest wall and diaphragm, which were removed at the same time. During the operation, several nodules were found in the chest wall and diaphragm muscle (Fig. 1L) and resected tumor (Fig. 1M).

**Table 1:** The results of flow cytometry of the patient in the Department of Cardiothoracic Surgery, Tianjin Medical University General Hospital.

	2020.9.29	2021.1.12	2021.1.21	2021.2.23	2021.3.4
CD20 lymphocytes	4.39	2.05	7.08	2.05	5.56
CD19	3.18	1.74	7.28	3.72	3.91
Breg/CD19+	6.17	2.88	5.73	7.37	7.67
B10/CD19+	4.94	5.77	14.06	3.69	4.89
Tfh	1.07	0.41	0.47	1.21	1.45
Tfr	0.59	1.09	0.82	0.34	2.25
Th17	2.25	4.15	2.63	0.81	2.52
Treg	3.19	3.39	4.61	4.63	3.65
CD3 + CD4+	26.59	30.99	37.29	26.7	18.76
CD3 + CD8+	61.8	59.12	51.9	62.82	74.70

Gross and microscopic histopathology revealed (Fig. 1K and M) reported that the patient’s anterior mediastinal mass, diaphragmatic nodules, and parietal pleural nodules were WHO class AB. After the operation, the patient recovered smoothly, and the laboratory related examinations approached normal (Fig. 2).

Due to the final diagnosis of thymoma is WHO class AB and stage IVA, the CAP regimen was given 4 times of adjuvant chemotherapy after the operation. During the chemotherapy, the glucocorticoid was gradual reduction. The blood picture was stable and the spleen continued to decrease under abdominal ultrasound. The symptoms of SLE were stable, and there was no impairment of liver and kidney function.

## 3. Discussion

At present, enhanced chest CT is the primary choice for preoperative examination in the evaluation of mediastinal tumors. Thymoma were more likely to be 40 to 60 years old, and their chest CT enhancement manifests as a smooth contour and a necrotic/cystic component of less than 50%.^[8]^ Compared with Masaoka stage I/II thymoma, stage III/IV thymoma tends to be larger in size, and imaging tumors with irregular shapes are more communal, and necrosis and calcification are often present. In addition, high-risk thymoma (type B2 or B3 thymoma) shows more irregular shapes and contours than low-risk thymoma (type A, AB, B1 thymoma), and is more prone to lobular or irregular contours. Areas of low attenuation and calcification.^[9]^

This patient’s chest CT showed a multilocular cystic solid mass with clear boundaries and a cystic component greater than 50% (Fig. 1D). Combined with the patient’s anemia, fever, and giant spleen and other clinical manifestations, clinically combined with SLE, the clinical diagnosis is considered as possible lymphoma. In the end, the tumor was punctured to achieve a pathological diagnosis and the diagnosis was thymoma. Therefore, for huge tumors of the anterior mediastinum, puncture is required first to obtain pathological diagnosis, which can guide treatment more accurately, and it also meets the recommendations of national comprehensive cancer network guidelines.^[10]^

Currently, the mechanism of thymoma with SLE is unclear. A variety of theories on the pathogenesis and development of autoimmune diseases in the context of thymoma have been proposed, mainly due to potential obstacles to normal thymic function, which in turn affects the process of positive and negative selection and central tolerance defects.^[2,3]^ These theories include escape theory or immature T cell theory hypothesizes, tumor theory or genetic theory, autoimmune regulatory factor gene mutation theory.^[2,3,11]^

The clinical manifestations of patients with thymoma combined with SLE are similar to those of patients with simple SLE. Analogous to simple SLE, a variety of antibodies can be detected in the serum of patients with thymoma combined with SLE, and there may be increased IgG and IgM, and decreased C3 and C4. The treatment strategy for patients with thymoma and SLE is the same as that for patients with SLE alone, and both are treated with glucocorticoids and hydroxychloroquine. Glucocorticoids are essential for quickly eliminating the autoimmune reactions that threaten target organ damage or even life-threatening.^[12]^

Glucocorticoids can result in a significant reduction in the number of Germinal Centers in the thymus. Glucocorticoids can reduce the proliferation of thymic follicles in the thymus, and also affect lymphatic tissue. This effect is especially important in female patients.^[13]^ Glucocorticoid therapy could change the number of high endothelial venules to normal by reducing the number of high endothelial venules and the chemokines that can attract T and B cells in the thymus produced, glucocorticoids may restrict the entry of activated cells into the thymus and prevent the formation of new germinal centers.^[14]^ Glucocorticoids can inhibit the expression of a variety of activated inflammatory genes, which encode cytokines, chemokines, adhesion molecules, inflammatory enzymes and receptors.^[15]^ Studies have shown that glucocorticoids have inhibitory effects on the epithelial and lymphocyte components of tumors.^[16]^ After glucocorticoid treatment, tumor epithelial cells were replaced by sclerotic fibrous tissue, accompanied by numerous foamy macrophages, hemorrhage and coagulative necrosis areas. Studies have demonstrated that after 17 patients with thymoma were treated with glucocorticoids before surgery, 8 patients with thymoma resolved.^[17]^

## 4. Conclusion

We experienced an extremely rare case of thymoma with SLE and immune-related anemia. Because it is sometimes difficult to differentiate thymoma and lymphoma, it possibly misleads us. There are many unclear features including clinical manifestations, pathology of this case, we hope to have further research to the disease.

## Acknowledgments

The authors thank the participants and contributors the case.

## Author contributions

All authors certify that this manuscript is a unique submission and is not being considered for publication by any other source in any medium. The manuscript has not been published, in part or in full, in any form.

**Conceptualization:** Xuesong Zhang.

**Data curation:** Xuesong Zhang.

**Formal analysis:** Ting Chen.

**Investigation:** Peng Zhang.

**Methodology:** Huayang Zhang.

**Supervision:** Peng Zhang, Yuan Chen.

**Validation:** Peng Zhang.

**Writing – original draft:** Huayang Zhang.

**Writing – review & editing:** Ting Chen.
